# Supplementary material for: Cumulative effects of weakly repressive regulatory regions in the 3’ UTR maintain PD-1 expression homeostasis in mammals
Source: Commun Biol. 2023 May 18;6:537. doi: 10.1038/s42003-023-04922-y (PMC10195876; doi:10.1038/s42003-023-04922-y)
Supplement: Supplementary file 2 — Supplementary Information [file 42003_2023_4922_MOESM2_ESM.pdf]

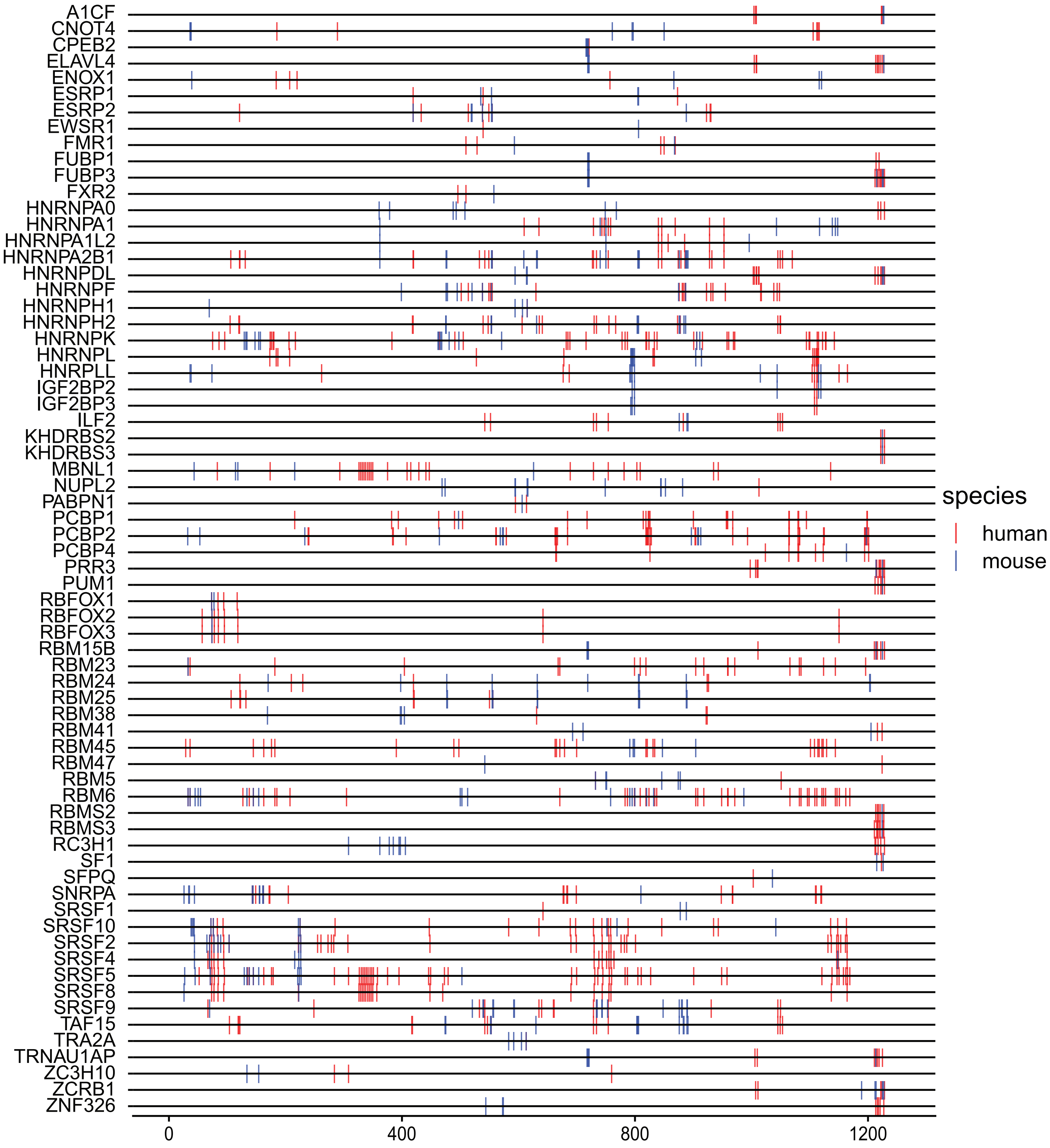

Supplementary Figure 1 All predicted RBP binding sites conserved between the human and the mouse PD-1 3' UTR

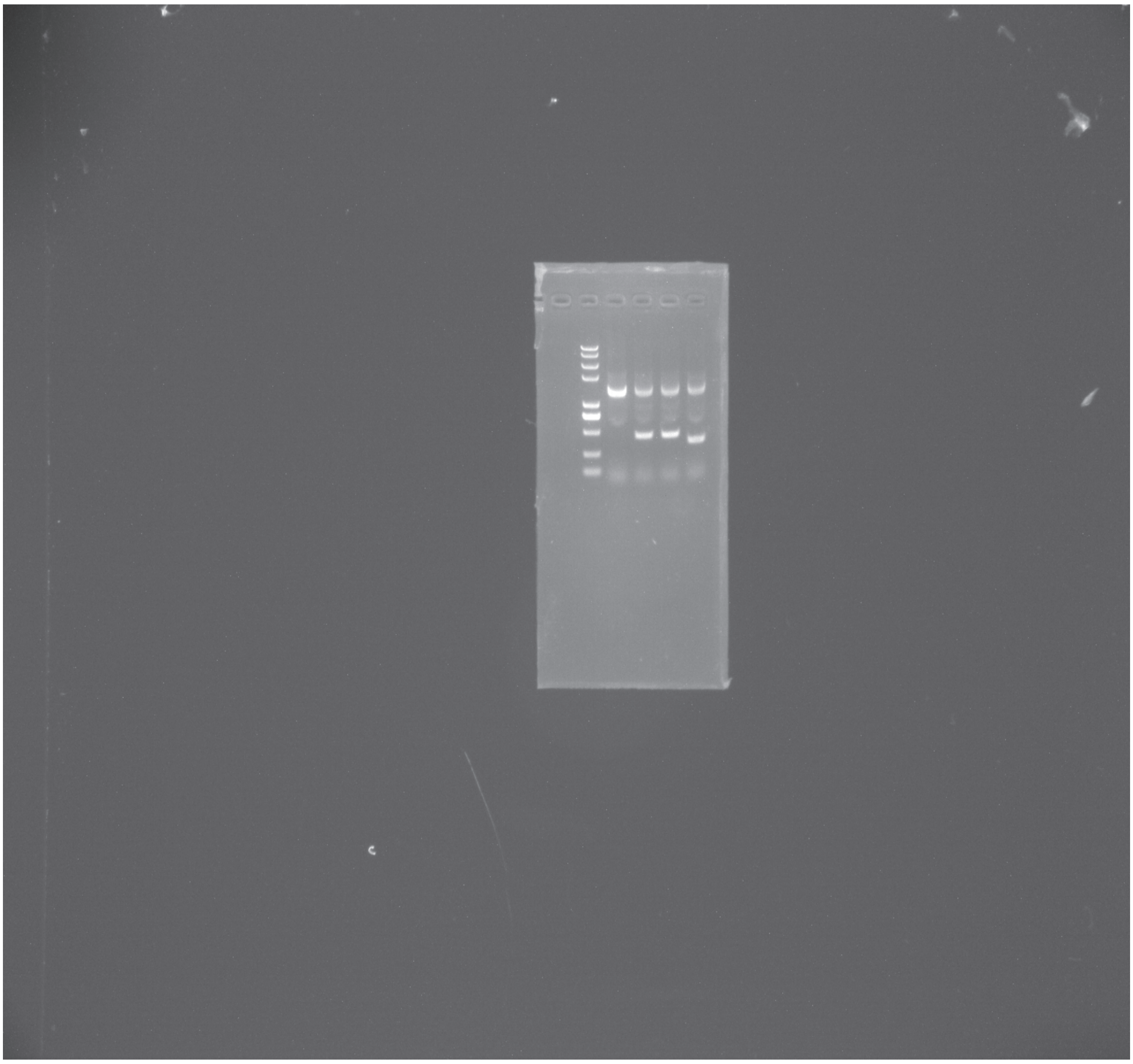

Supplementary Figure 2. The uncropped and unedited PCR gel picture related to Figure 2a.
