# Supplementary material for: Cumulative effects of weakly repressive regulatory regions in the 3’ UTR maintain PD-1 expression homeostasis in mammals
Source: Commun Biol. 2023 May 18;6:537. doi: 10.1038/s42003-023-04922-y (PMC10195876; doi:10.1038/s42003-023-04922-y)
Supplement: Supplementary file 3 — Description of Additional Supplementary Files [file 42003_2023_4922_MOESM3_ESM.pdf]

Description of Additional Supplementary Files

File name: Supplementary Data 1

Description: The conservation scores of coding regions and 3' UTRs of 21,050 genes of 99 vertebrates.

File name: Supplementary Data 2

Description: All oligonucleotide primers used in this work.

File name: Supplementary Data 3

Description: Numerical source data for graphs
